# Roles of the Different Sub-Regions of the Insular Cortex in Various Phases of the Decision-Making Process

**DOI:** 10.3389/fnbeh.2015.00309

**Published:** 2015-11-25

**Authors:** Vita Droutman, Antoine Bechara, Stephen J. Read

**Affiliations:** Department of Psychology, University of Southern CaliforniaLos Angeles, CA, USA

**Keywords:** insula, decision-making, risk, uncertainty, evaluation, urge generation, error processing, cognitive-control

## Abstract

This paper presents a coherent account of the role of the insular cortex (IC) in decision-making. We follow a conceptualization of decision-making that is very close to one previously proposed by Ernst and Paulus (2005): that the decision process is a progression of four phases: (1) re-focusing attention; (2) evaluation; (3) action; and (4) outcome processing, and we present evidence for the insula’s role in all these phases. We review the existing work on insula’s functional anatomy that subdivides the IC into posterior, dorsal anterior and ventral anterior regions. We re-map the results provided by the existing literature into these subdivisions wherever possible, to identify the components’ role in each decision making phase. In addition, we identify a self-regulating quality of the IC focused on harm avoidance.

The insular cortex (IC) originally was thought to be “a portion of the visceral brain” and “was not even worthy of a number” on Brodmann’s map (Craig, 2010b, p. 395). As our understanding of its functionality has progressed, the insula has become known as a center of interoception, emotion and awareness (Craig, 1996, 2009a,b, 2010a,b). Recently, its role in attention, executive functioning and decision-making have also come to light. Evidence of the insula’s activation, at first unexpected and un-explained in many unrelated studies, finally reached critical mass and the insula has become a focus of exploration. It has recently been noted that the IC is one of the few neural components that is consistently activated across thousands of studies (Duncan and Owen, 2000; Nelson et al., 2010; Yarkoni et al., 2011; Chang et al., 2012). Many of these accounts identify the IC’s involvement in different aspects of decision-making, such as anticipation of gain and losses (Knutson and Greer, 2008), and urge processing (Garavan, 2010), among many others. Unlike its role in interoception, emotion, and awareness, however, the role of the insula in decision-making has not been fully mapped out. This paper aims to present a coherent account of the IC’s role in decision making in terms of a combination of all the known aspects of its function integrated together into a comprehensive picture.

One of the most influential conceptualization of the neurocognitive processes sub-serving decision-making was provided by Ernst and Paulus (2005). They divided the decision process into three phases (evaluation, action, and outcome). We follow this conceptualization closely except that we add one earlier phase, i.e., re-focusing attention. We examine the role of different sub-regions within the insula in the decision making process, which is viewed as a progression of four phases: (1) re-focusing attention; (2) evaluation; (3) action; and (4) outcome processing. Specifically, we present evidence on the role of different sub-regions of the insula in each of these phases. In addition, we identify a self-regulating quality of the IC focused on harm avoidance.

## Structural and Functional Anatomy of the Insular Cortex

Three major subdivisions of the IC have been identified based on its internal structure both in humans (Morel et al., 2013) and non-human primates (Mesulam and Mufson, 1982): (1) the granular insula, which is located in the posterior dorsal portion of the IC; (2) the agranular insula located in the anterior ventral portion of the IC; and (3) the dysgranular insula—a large band occupying the middle portion of the IC. Mufson and Mesulam (1982) used axonal transport methods to examine structural connectivity of the IC in rhesus monkey and found both commonality and differences in projections from both anterior (agranualar and dysgranular subdivisions) and posterior IC. Projections from both directions reached orbitofrontal cortex (OFC), anterior cingulate cortex (ACC), temporal and parietal lobes; however, only anterior IC projections were found in prepiriform olfactory cortex. Stronger projection to OFC and temporal lobes was identified from the anterior IC and to ACC and parietal lobe from the posterior IC. Difference in the projections towards the IC were also found. Specifically, projections from ACC were found in all areas of IC, but projections from OFC were mostly directed to AIC.

Structural connectivity examinations of the IC in humans is limited to two accounts (Cloutman et al., 2012; Jakab et al., 2012). Jakab and colleagues examined voxel based structural connectivity patterns for each voxel within the IC and identified three insular segments based on the pattern’s similarity: anterior, dorsomedial and posterior. Cloutman and collegues had convergent findings with an ROI based approach; tracing connectivity of seven anatomically based ROIs resulted in identification of three similar segments.

**Figure 1:**
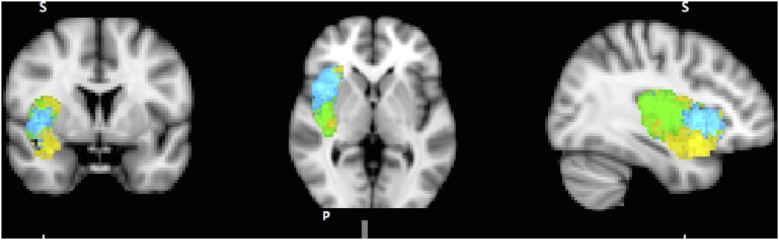
**Sub-regions of the Insula: posterior (green), dorsal anterior (blue) and ventral anterior (yellow).** Sub-region masks provided by Chang et al. (2012) downloaded from NeuroVault.org.

A dozen or more analyses of the IC’s functional parcellation have been performed, and they have identified two (Cauda et al., 2012) to four components (Kurth et al., 2010). The most convergent account, reached by several methodologies, including functional connectivity analysis (Chang et al., 2012) and meta-analysis of neuro-imaging data (Wager and Feldman Barrett, 2004; Mutschler et al., 2009; Kurth et al., 2010), identifies three subregions within the IC (Figure 1): posterior insular cortex (PIC) responsible for sensorimotor processing (Craig, 2002; Wager et al., 2004), dorsal anterior insular cortex (dAIC) involved in cognition (Dosenbach et al., 2006; Eckert et al., 2009), and ventral anterior insular cortex (vAIC) associated with social-emotional processing (Sanfey et al., 2003; Chang et al., 2012).

Structural and functional IC parcellations align only in the posterior region. Additional experimental work is necessary to reconcile the inconsistencies between functional and structural connectivity of the AIC. Since functional parcellation literature is more prevalent and provides better spatial resolution and point-to-point components identification, we will focus on its findings for the rest of this paper.

In the most recent paper in this line of work, Chang et al. (2012) used a multi-modal approach to parcellation by combining functional connectivity analysis with newly developed meta-analytical methods in order to address both consistency and specificity of the insular networks activity. They first performed resting state functional connectivity analysis that confirmed the tripartite division of the IC into posterior, dorsal anterior, and ventral anterior sub-regions identified in the prior literature described above. The novelty of their approach, however, is the identification of broader networks that are co-activated with the IC sub-regions, first from their own functional connectivity data, and then validated by an innovative meta-analytical method involving the complete NeuroSynth database (Yarkoni et al., 2011; all 4393 studies available at the time of analysis). Although the networks revealed by both methods were not identical, there is significant overlap between the findings. Specifically, identified in both analyses, the ventral anterior network included links primarily to emotion related areas, namely the amygdala, ventral-tegmental area (VTA) and lateral OFC; the dorsal anterior network included links to cognitive control related areas, namely the ACC and dorsolateral prefrontal cortex (DLPFC); and posterior network included links to the supplementary motor area (SMA) and somatosensory cortex (for full networks identified by both methods see Table 1).

**Table 1:** **Defining neural networks consistent with and specific to insula subdivisions into posterior, dorsal anterior and ventral anterior components**.

	Network components	Network component unique to	Network components unique to	identified by both analysis	functional connectivity analysis	NeuroSynth meta-analysis
Ventral-Anterior	amygdala, ventral tegmental area (VTA),	superior temporal sulcus	ventral striatum, temporal poles,
	posterolateral orbitofrontal cortex		medial prefrontal cortex (MPFC)
Dorsal-Anterior	anterior cingulate cortex (ACC) and		dorsal striatum, temporo-parietal
	dorsolateral prefrontal cortex (DLPFC)		junction (TPJ)
Posterior	supplementary motor area (SMA),		posterior temporal lobes,
	somatosensory cortex		right hippocampus, rostral ACC

*Networks identified by functional connectivity analysis and by NeuroSynth meta-analysis (Chang et al., 2012*.

The authors further examined the functional focus of the identified networks, utilizing hundreds of forward and reverse inference^1^ meta-analysis maps available in NeuroSynth. Their findings indicated that the dorsal anterior insular network focused on executive control and higher cognition, the ventral anterior insular network was associated with emotion, chemo-sensation and autonomic functioning, and the posterior insular network was primarily connected with pain, sensorimotor and language processing.

With the above high-level anatomical map in mind we can now focus on more detailed accounts of insula’s role in different phases of decision-making. To incorporate the parcellation work into this review we attempted to remap prior published findings (typically identified simply as insula, or AI or PI in the published articles) into the sub-regions proposed by Chang et al. (2012)^2^^,^^3^ using their coordinate maps. Our results are somewhat limited because* a priori* ROIs were used in some papers, thus potentially excluding activations in the areas important for this analysis; also, some works did not publish coordinates at all, but only included images while discussing their findings. In spite of these limitations, we believe that the remapping results offer valuable insights and, to the extent possible, we will try to tie the review to specific functional regions of the insula. All through the rest of the paper the differentiations of dorsal anterior, ventral anterior and posterior insula (while the existing findings are discussed) are the results of our remapping exercise (see Table S1 in Supplemental Materials for the full remapping list). There were only a few exceptions that will be noted, when the original authors identified the sub-regions. The portions of the paper that simply refer to “anterior insula” or “insula” discuss the findings for which coordinates were not available, and thus no greater anatomical specificity was possible.

## Role of the Insular Cortex in Re-Focusing Attention

**Figure 2:**
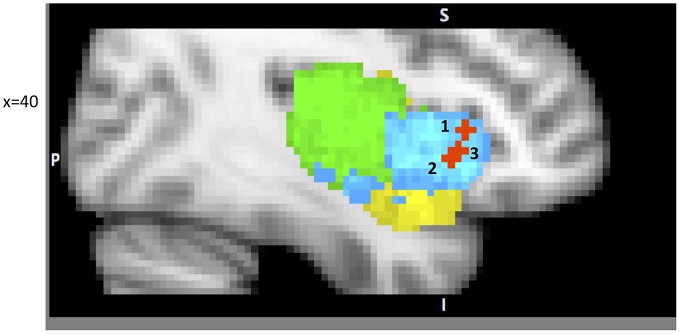
**A sample of studies addressing the IC’s role in Attention Focusing Phase (for illustration purposes only).** Sub-regions of the Insula: posterior (green), dorsal anterior (blue) and ventral anterior (yellow). 1 – IC as part of the CCN in Cole and Schneider (2007) (38, 22, 5); 2 – IC as part of task-set system in Dosenbach et al. (2006) (40, 19, −3); 3 – IC as part of Salience Network (SN) in Sridharan et al. (2008) (34, 26, −6).

A growing body of research recognizes the insula’s role in attention and executive functioning (Dosenbach et al., 2006; Ploran et al., 2007; Tops and Boksem, 2011; among many others). We will focus on several reports that emphasize the role of the IC as a part of specific attention processing networks (Figure 2). Cole and Schneider propose the existence of a Cognitive Control Network (CCN), represented by dAIC, ACC, DLPFC, inferior frontal junction (IFJ), dorsal pre-motor cortex (dPMC), and posterior parietal cortex (PPC; 2005). They present converging evidence from multiple methods including: co-activation during task performance, high functional connectivity at rest and during the task, and consistently higher correlations within the CCN than the rest of cortex. They first identified components of the proposed CCN by whole brain analysis of a visual search task, designed specifically to isolate cognitive control from working memory processing. High inter-component correlation between CCN regions during task (average 0.76) and at rest (average 0.74) provides additional evidence in support of their hypothesis. This work constitutes a persuasive argument for the existence of a CCN, and is consistent with the parcellation map presented above Chang et al. (2012), since the DLPFC, ACC and dorsal anterior insula comprise the dorsal anterior insular network responsible for attention and cognitive function.

Dosenbach et al. (2006) offer a similar but narrower perspective by classifying dAIC and dACC as a “task-set system” that is activated by a start-cue and sustains activations during task performance (in 10 separate tasks). This account is consistent with Nelson et al. (2010) who identify the most anterior portion of IC, in coordination with ACC, as associated with “capture of focal attention” (p. 669). Building upon these findings Menon and Uddin (2010) propose the existence of a salience network (SN), consisting of the dAIC and ACC, focused on identifying the most relevant stimuli or events (either internal or external) and switching between other large networks to facilitate initiation of attention. In another paper from this lab (Sridharan et al., 2008) the authors explored the temporal dynamics and causal direction between nodes in the SN, the central executive network (CEN) and the default mode network (DMN). First, they address the issue of whether there is one larger CCN network as proposed by Cole and Schneider or whether there are two separate but coordinated networks, SN and CEN (DLPFC and PPC). Their findings point to the latter solution. Although SN and CEN components are often co-activated, independent component analysis identified two distinct networks. Moreover, by estimating onset and peak latencies of the BOLD response, the authors show that activation in SN precedes that in CEN and DMN. Finally, the evidence from Granger Causality Analysis indicates that right dAIC has a key role in switching between CEN and DMN. These findings were consistent across different stimulus modalities (visual, auditory and resting state).

## Role of the Insular Cortex in Stimulus Evaluation

We will next examine the role of IC in the evaluation phase of the decision process. The high-level (or more general) role of IC in the evaluation stage of decision-making was outlined in the Somatic Marker Hypothesis (SMH) that “provides a systems-level neuroanatomical and cognitive framework for decision-making and its influence by emotion” (Bechara and Damasio, 2005, p. 336). During the evaluation of complex stimuli, previously learned somatic state patterns triggered by amygdala or vmPFC are either activated directly in the IC, or are related back to the IC after having been re-enacted first in the body (Bechara and Damasio, 2005). These “somatic markers” play a key role in evaluation, often biasing response options and action plans. It is important to note that this evaluation step may be conscious (i.e., accompanied by a certain subjective feeling), or, most often, subconscious (i.e., implicit and without any accompanying conscious feelings). SMH originated from behavioral and psychophysiological examination of patients with lesions in ventromedial PFC. Its neural framework was supported more recently (Lawrence et al., 2009; Li et al., 2010) using functional neuroimaging work, which further confirmed the AI’s role (among all the other components in the neural framework) in the evaluation step of the decision-making process. Specifically, both dorsal anterior and ventral anterior sub-regions were active for risky-decisions, but only dAI activation positively correlated with overall game score (Lawrence et al., 2009). It is possible that the dAI’s role in coordinating attention (discussed above) was responsible for this effect. Game score also positively correlated with activation in medial orbitofrontal cortex (OFC). This finding resonates well with lesion work that identified the OFC as a critical region in risk estimation (Weller et al., 2007), and the insula as important in risk adjustment (Clark et al., 2008).

### Evaluation of Valence and Arousal

The more specific application of the SMH may begin with the role of the AI in evaluating valence and arousal. Important insights come from the work of Berntson et al. (2011) who examined evaluative processes in patients with insula lesions. The study compared patients with lesions in the IC with two control groups: patients with amygdala lesions and patients with lesions in areas that spare both insula and amygdala. The participants evaluated positive, negative and neutral images from the International Affective Picture System (IAPS; Lang et al., 1999) on two-dimensional valence and one-dimensional arousal scales. Patients with insula damage differed from patients in the two other groups in both positive and negative valence ratings, showing smaller increment for both positive and negative images. Similarly, patients with insula damage demonstrated attenuated arousal to positive and negative images compared to both control groups. Patients with amygdala damage only indicated reduced arousal to negative images compared with the second lesion contrast group patients. This finding confirms a broader role for the IC in the evaluation process that impacts both arousal and valence judgments. Neuroimaging findings also support this hypothesis. Specifically, increased activation in dAIC and vAIC was evident when evaluating the stimuli associated with potential gains or losses (Knutson and Greer, 2008). These increases in activation correlated with subjective affective arousal (both positive and negative) indicating that AIC may be sensitive to arousal in general, or it may respond to both positive and negative valence. We interpret these findings as a result of the AIC being an integrative interoceptive site connecting autonomic, affective and cognitive processing (Craig, 2009a; Critchley, 2009), and they can be viewed as consistent with the SMH framework. These findings are also consistent with the SN hypothesis.

### Evaluation of Magnitude, Variance and Skewness

However, we also find very specific activity of the AIC, which indicates sensitivity to different characteristics of the stimulus, such as magnitude, variance and skewness that goes far beyond simply identifying salience. Several studies find evidence that the anterior insular cortex^4^ tracks the magnitude of a reward, at least in case of gains, with stronger activation corresponding to larger reward (Paulus and Frank, 2006; Smith et al., 2009). This finding was confirmed and extended in a recently conducted ALE meta-analysis of the fMRI literature on financial decisions (Wu et al., 2012). The authors found that dorsal anterior insula is sensitive to the magnitude of the reward (high vs. low mean contrast), and that both dorsal and ventral anterior insula are sensitive to variance (high vs. low variance contrast).

Even more intricate specialization is necessary to differentiate reward skewness. The following study illustrates how reward skewness sensitivity is evaluated (Burke and Tobler, 2011). The researchers used a set of abstract images, each associated with a ternary lottery. At the start of the experiment and prior to the scanning session, participants learned image-reward set associations and were instructed that during the lottery all rewards within the set have an equal probability of being selected. Each set of interest had the same mean and variance, but the skewness of the distribution of rewards differed between positive (19, 21, 65), negative (5, 49, 51) and zero (9, 35, 61). During the scan, participants played the set of lotteries represented by previously learned images. The outcomes of the lottery trials were not given to the participants until the end of the session so as not to confuse the anticipatory vs. outcome related activation. The analysis of activation revealed increased activation in vAIC with increasing skewness. When behavioral preference for skewness was included in the analysis, it revealed that insula tracking was correspondent with objective skewness (personal preference for certain skewness did not effect the IC activation). Corroboration of this finding (increased activation in vAIC with increased skewness) was presented in two other studies (Symmonds et al., 2011; Wu et al., 2011).

### IC Roles in Decisions Under Uncertainty and Risk

The insula has also been identified in prior work as a key structure in the neural system involved in decision-making under uncertainty (Weller et al., 2007). Altered decision-making under uncertainty involving both risky gains and risky losses was observed in patients with insula lesions (Weller et al., 2009). Specifically, patients with insula damage were less sensitive to differences in expected value (EV) between the options. Converging findings come from functional neuroimaging studies. Increased anticipatory dAIC activation (Paulus and Frank, 2006; Knutson et al., 2007; Levin et al., 2012) was evident to potential gains and potential losses. Moreover, this increase in anticipatory affect strongly influenced the decisions’ outcome. Specifically, the evidence from purchasing paradigm studies shows that anticipatory vAIC activation while viewing the prices predicted with high accuracy that subjects would be less likely to buy a product (Knutson et al., 2007; Grosenick et al., 2008). In a gambling paradigm, dAIC activation was predictive of selection of a safe choice (Kuhnen and Knutson, 2005). To summarize, this work offers evidence that anticipation of uncertain positive or negative rewards elicits activation in regions of the dorsal and ventral AI, which correlates with self-reported arousal and in the case of anticipated negativity (high price or risk), predicts negative evaluation of outcome.

The question of whether this increased activation simply indicates reactivity to reward/loss or whether the insula plays a role in tracking uncertainty was addressed by Huettel et al. (2006), who showed that dAIC activation related to decision ambiguity.

Finally, Preuschoff et al. (2006, 2008) uncovered another specific function of the AIC, tracking of risk prediction and risk prediction errors. Their findings indicate the existence of a risk prediction signal, defined as risk associated with uncertainty of outcome and measured by reward variance, encoded by dAIC^5^. Risk prediction errors that arise when risk prediction is misjudged and that may be used for improvement of risk prediction in the future, are tracked by vAIC.

**Figure 3:**
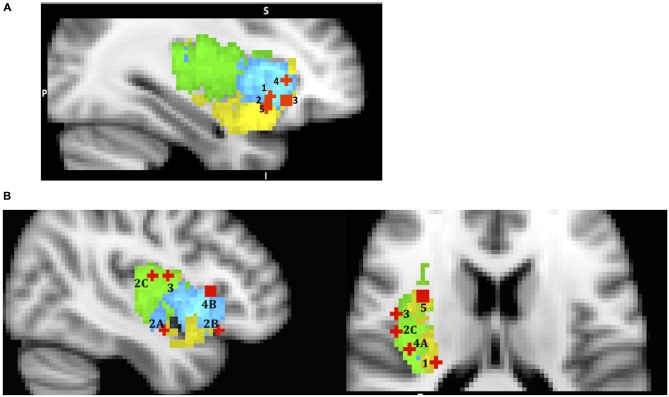
**A sample of studies addressing the IC’s role in Evaluation Phase (for illustration purposes only).** Sub-regions of the Insula: posterior (green), dorsal anterior (blue) and ventral anterior (yellow). IC activations related to **(A)** 1 – mean difference in Wu et al. (2012) (36, 19, −5); 2 – variance in Wu et al. (2012) (36, 16, −9); 3 – skewness in Wu et al. (2011) (33, 26, −7); 4 – risk prediction in Preuschoff et al. (2008) (37, 25, 1); 5 – risk prediction error in Preuschoff et al. (2008) (36, 17, −11); **(B)** (urge processing) 1 – smoking cue reactivity in Engelmann et al. (2012) (−28, −26, 14); 2 – alcohol cue reactivity in Myrick et al. (2004) (A) (41, −5, −14); (B) (47, 22, −13) C in Tapert et al. (2004) (47, −11, 15); 3 – cocaine cue reactivity in Garavan et al. (2000) (45, −4, 14); 4 – consciousness of thirst in Egan et al. (2003) (A) (39, −20, 18) and (B) (43, 19, 6); 5 – sexual arousal in Safron et al. (2007) (34, 5, 16).

In summary, the reviewed studies support the view that the AIC plays an important role in evaluating both positive and negative stimuli; it is responsible for coordinating with other brain areas or networks when necessary; and it is sensitive to uncertainty, value, variance, reward skewness and risk prediction (Figure 3A). The evidence suggests that the dorsal anterior sub-region’s role is quite wide and general (evaluating gains and losses, uncertainty and risk processing), and this is consistent with the SN hypothesis. However, the ventral anterior sub-region’s role is more specialized, such as evaluating variance, skewness of potential rewards and encoding reward prediction errors.

### Urge Generation and Neural Processing

The relevance of this topic to the current discussion stems from the fact that urges are part of the evaluation phase of the decision process because they arise when confronted with certain stimuli and during their evaluation. We can look at urges as another manifestation of “somatic markers” as described by the SMH. Since urges are best triggered and identified in cases where substances are involved (for example, the urge to smoke or to use a drug), we begin our discussion by describing the neural processing of “urge” proposed by studies on drug use. During drug use, interoceptive signals of physiological sensations associated with the hedonic experience first reach the posterior insula, and then are transmitted to the anterior insula, where they reach awareness and are committed to memory (Naqvi et al., 2014, p. 7), thus creating somatic marker representations. Later, when confronted with drug related stimuli, the previously stored somatic pattern associated with the experience of the drug is recalled by the AIC, and this in turn activates the drug-seeking goal. In addition, the generation of an urge, (such as when you deprive someone of a cigarette), can magnify the value of the somatic marker representation (i.e., the conscious feelings of craving or urges may turn the volume up) hence amplifying the importance of the drug seeking goal. So it is not surprising that many neuroimaging studies have found increased activation of the IC in subjects exposed to drug related stimuli, and that IC activation was correlated with conscious experience of the urge to take drugs (Naqvi and Bechara, 2009).

Naqvi and Bechara (2009) list 16 neuroimaging studies in their review that find insula activation to cigarettes, alcohol, cocaine and heroine related cues, and many of these studies show correlation of insula activity with subject’s ratings of the urge. Based on this evidence, along with findings in the animal literature, researchers propose that the insula’s key role in drug addiction is in encoding “a representation of the interoceptive effects of drug use that become activated when an addicted individual is exposed to environmental drug cues” (p. 61). Our re-mapping effort yielded activation in vAIC & PIC for cigarettes (Engelmann et al., 2012), vAIC, dAIC (Myrick et al., 2004) and PIC (Tapert et al., 2004) for alcohol, and PIC for cocaine (Garavan et al., 2000) cues (Figure 3B). Although it is possible that different substances trigger activations in different sub-regions of the insula, it is more likely that all sub-regions are involved, but different study designs allow only some of them to be detected. Therefore a likely explanation for these findings is that when a drug related stimulus is presented, its somatic marker is evoked by dAIC, represented through vAIC, which, in turn, initiates the physical sensation of craving that is processed by PIC. This process reverses the pathway involved during original drug exposure. This role of the posterior insula echoes the suggestion that Naqvi et al. (2014, p. 5) made while discussing the rodent lesion studies’ literature, that “the posterior insula is necessary for registering the reinforcement value of drugs”. However, more research is necessary to confirm such a mechanism in humans.

Mounting evidence confirms the key role of the insula in substance addiction. Furthermore, evidence exists that the insula’s role is not limited to addiction, but it is characteristic of urge processing more generally. The IC is found to be involved in urges related to hunger and thirst (Tataranni et al., 1999; Del Parigi et al., 2002; Egan et al., 2003), erotic stimuli (Gizewski et al., 2006; Safron et al., 2007), and even itching and scratching (Vierow et al., 2009). Most of these studies^6^ identified increased activation both in posterior and dorsal anterior insula. Egan et al. (2003) focused on the temporal dimension and found increased activation in posterior insula corresponding to onset of thirst, and in the dorsal anterior region found activation during maximum thirst increase. This finding suggests that posterior insula is the first to receive interoceptive input, and dorsal anterior area is responsible for initiating a response to this homeostatic imbalance. We maintain that the original role of the IC in urge processing was evolutionary-protective and directed toward satisfaction of primary biological needs like nutrition, hydration, and reproduction; focused on survival and the maintenance of homeostasis (Naqvi and Bechara, 2010; Naqvi et al., 2014). It was later “chemically hijacked” by the overwhelmingly reinforcing nature of addictive substances.

## Role of the AIC in Action Selection

The role of the IC in action selection is evident from studies that examine intentional acts. Intentional acts differ from stimuli-driven behaviors in that the environment does not trigger them, but rather they are internally motivated and generated (Brass and Haggard, 2010). Three different aspects of intentional action have been investigated. A set of EEG^7^ studies focused on the “when” component, where participants had a choice of timing of the action compared to the trials where timing was controlled by external stimuli (Ball et al., 1999; Jenkins et al., 2000; Cunnington et al., 2002; Wiese et al., 2005). Another set of EEG studies focused on the “what” component, or selecting between several alternatives, often represented by laterality (which button to press; Lau et al., 2004; van Eimeren et al., 2006; Mueller et al., 2007).

The evidence of the AIC’s role in “what” and “when” components is mixed. However, Jenkins et al. (2000) found strong bilateral dAIC activation when comparing self-initiated key presses with externally triggered ones. Mueller et al. (2007) found activation of the right vAIC and left dAIC when comparing free choice and stimulus-driven right or left responses.

The third aspect of action selection is “whether” to act or not, where participants are given freedom to decide not to perform the planned behavior (Brass and Haggard, 2007; Kühn et al., 2009). Lesion studies and structural connectivity work implicate AIC as a critical player in inhibitory control (Hodgson et al., 2007; Forstmann et al., 2008). Brass and Haggard (2007) study on intentional action and stopping found activation in the vAIC for intentional stopping (subjects had a choice when to press the button but were instructed to refrain from pressing the key on some trials of their choice). In another study the authors compared activation during a decision to cancel the action with instructed action and instructed stopped action (Kühn and Brass, 2009). Activation in the AIC did not differ between a decision to go and a decision to stop, but comparison of a decision to stop and instructed stop highlighted activation in the dAIC.

**Figure 4:**
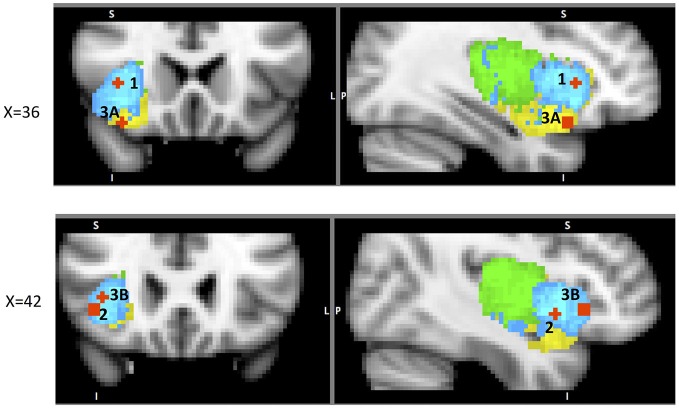
**A sample of studies addressing the IC’s role in Action Phase (for illustration purposes only).** Sub-regions of the Insula: posterior (green), dorsal anterior (blue) and ventral anterior (yellow). IC activation related to 1 – when to act in Jenkins et al. (2000) (38, 22, 2); 2 – what action to choose in Mueller et al. (2007) (43, 10, −6); 3 – whether to act **(A)** in Brass and Haggard (2007) (35, 19, −17) and **(B)** in Kühn and Brass (2009) (42, 24, −4).

This literature suggests that the AIC is particularly sensitive to voluntary action or decision to act and that its involvement corresponds to the effort needed both for action and the decision to act. The evidence discussed above suggests that dAIC is involved in “what”, “when” and “whether to act or not” aspects of intentional action, whereas vAIC is involved in the inhibition of an intentional action (Figure 4).

## Role of the Insular Cortex in Outcome Processing

### Error Awareness and Post-Error Correction

The last phase of a decision process is the post decision stage, and outcome evaluation. Consistent with its role as the “center of awareness” (Craig, 2009b), the insula has been shown to be a key neural structure responsible for error awareness. Meta-analysis of 55 neuro-imaging studies found consistent anterior insula activation during error commission, and also in cases where performance monitoring was required (Klein et al., 2007). More specifically, contrasting aware errors with unaware errors yielded increased activation in dAIC bilaterally (Klein et al., 2007). In their review, Ullsperger et al. (2010) discuss an integrated role of the AIC and autonomic nervous system (ANS) in error awareness. They focus on pupil diameter (PD), as an index of autonomic arousal connected to action accuracy that was correlated with AIC’s activity during error processing (Critchley et al., 2005). PD change from before to after aware errors co-varied with increased activation in AIC and deactivation in the DMN (Ullsperger et al., 2010). This interpretation is not surprising since AIC activation has been identified as a neural correlate of ANS activity by (Craig, 2002, 2009b). These findings also point to the aforementioned SN, and suggest that AIC recruits necessary resources after an error has been detected (Ullsperger et al., 2010).

**Figure 5:**
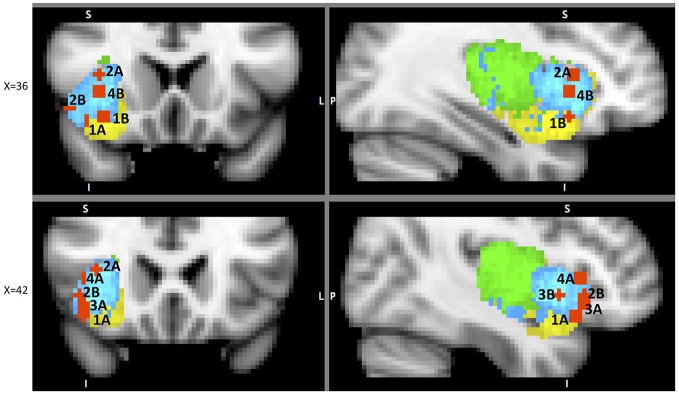
**A sample of studies addressing the IC’s role in Outcome Processing Phase (for illustration purposes only).** Sub-regions of the Insula: posterior (green), dorsal anterior (blue) and ventral anterior (yellow). IC activations in reaction to 1 – error awareness **(A)** in Klein et al. (2007) (41, 23, −14) and **(B)** in Harsay et al. (2012) (34, 18, −12); 2 – post error slowing in Li et al. (2008), **(A)** (36, 20, 8) and **(B)** (44, 24, −4); 3 – non-reciprocated cooperation in Rilling et al. (2008a) (41, 25, −8); 4 – harm prevention **(A)** in Kuhnen and Knutson (2005) (42, 23, 5) and **(B)** in Campbell-Meiklejohn et al. (2008) (36, 18, 0).

Of particular interest in this regard is the study of the AIC’s functional connectivity during error awareness (Harsay et al., 2012). In this study, the same group of participants performed two unrelated tasks: an error awareness task (with self-evaluation after every trial) and an oddball task focused on identifying BOLD response to salient events. Experimenters then evaluated potential overlap in activation between aware errors (contrast: aware errors—non-aware errors) and oddball event identification. Group level spatial overlap analysis identified right dAIC, vAIC, dACC, somatosensory cortex, precentral gyrus (frontal eye-fields), thalamus, and brainstem common to both processes. The authors inspected the spatial spread of activation in AIC and found interesting topographic differences: error awareness increased activation localized to vAIC, but salience processing related activation spread across both dAIC and PIC with maxima in dAIC. They also found high correlations between hemodynamic responses in anatomically defined AIC ROI between the two processes. While re-mapping their results we noticed that one of the two foci in the IC that showed significantly higher activation during aware than unaware errors was located in vAIC and the other in dAIC. This suggests somewhat distributed neural processing of error awareness. Similar activations for salient events identification and error awareness advocate for a SN model of AIC’s role in error awareness that is likely supported by dAIC. However, similar to what we have seen in the evaluation phase, this doesn’t represent the whole picture: vAIC is also a key component involved in error awareness. We can speculate, that vAIC identifies error related events and then “alerts” dAIC, together with the SN, to deal with these conflicts (Figure 5). This explanation makes sense especially in light of post-error corrective behavior, which is supported in large part by dAIC, as we will discuss in the following section. However, extensive additional research is necessary to substantiate such claims.

The major significance of error-awareness is the post-error corrective behavior it causes. Published studies show that aware errors result in slower response time (RT) immediately following correct trials (Nieuwenhuis et al., 2001; Rilling et al., 2008a). An fMRI study focused on mapping neural components responsible for post-error correction found increased activation in the dAIC along with the dorsolateral prefrontal cortex (DLPFC), and the fronto-polar cortex (FPC) when comparing post-error trials with increased RT to the ones where the RT was not increased (Li et al., 2008)^8^. Further functional connectivity analysis revealed that all three areas were strongly correlated.

### Role of the IC in the Outcome Phase of Social Decisions

Post-error slowing is one of the IC’s several functions that are focused on harm-reduction and harm avoidance. We now explore other known representations of this protective purpose of the IC. One such example is its role in social decisions. Social decision-making is usually evaluated with the prisoner’s dilemma (PD), the trust game (TG), the ultimatum game (UG) or the dictator game (DG), each involving a decision to trust or not to trust the partner on each trial. In PD, participants make simultaneous decisions whether or not to trust each other without knowing their partner’s respective choice. In this task non-reciprocated cooperation was associated with activation in both dAIC and vAIC in the cooperator (Rilling et al., 2008a). This finding is not surprising and can be explained by the expression of negative emotions toward non-reciprocating partner. The areas of activation from the above analysis^11^ were used to define ROIs to examine functional connectivity during the trials with non-reciprocated cooperation. The results indicate that functional connectivity between AIC and LOFC predicts defection in future interactions with the same non-reciprocating partner. The authors suggest that LOFC, AIC, amygdala and hippocampus constitute a network specializing (not exclusively) in violations of social contracts^9^ (Rilling et al., 2008b); this network is intriguingly similar to the one identified by Chang et al. (2012) as the ventral anterior insular network (see Table 1). We suggest that the above results are another illustration of the resource-recruiting protective function of the IC, similar to the one we observed in post-error slowing (Figure 5).

A similar focus is evident in the UG, a bargaining paradigm where a reward amount is to be divided between the two players. Here, the participant is offered a certain ratio by another player and can either accept or reject it; in the case of rejection, neither player receives money. In a neuroimaging study, Sanfey et al. (2003) found increased activation in the AIC^10^, DLPFC and ACC when comparing unfair and fair offers. Moreover, the activation in AIC scaled with the magnitude of unfairness and unlike the activation in DLPFC and ACC, strongly correlated with the rate of rejection of the unfair offers. The authors also found that for the unfair offers that were rejected the activation in AIC was higher than activation in DLPFC and* vice versa*. They explain this finding by suggesting that DLPFC supports the goal of accumulating the maximum amount of money and the AIC reflects the social response to the offer.

### IC’s Role in Harm Prevention

**Figure 6:**
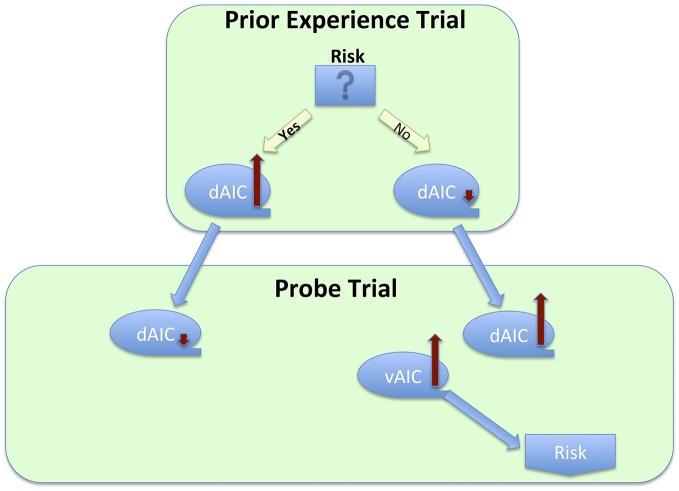
**Process flow of a series of decisions between risky and safe option, and corresponding changes in activation across sub-regions of the insula cortex**.

Finally, the preventive function of the IC is linked to its role in risk or uncertainty related decisions. Several research groups have examined an effect of prior experience on subsequent trials and have found a risk decreasing effect, mediated by the IC activation in a preceding trial. Specifically, Xue et al. (2010) examined the effect of prior risk on subsequent decisions. They used a version of the CUPS task, a gambling task, in which participants are presented with a series of mixed gambles of variable EV; each trial requires a decision whether to accept or reject the gamble. The task was modified to strategically group trials in pairs, so that in the first trial in the pair (called *prior experience trials*) participant’s choice to gamble or not was anticipated with high certainty due to the EV being either positive or negative. It was followed by the trial with *EV* = 0 (called *probe trials*), thus allowing for the evaluation of EV-independent risk taking. This elegant design enabled the authors to examine the effect of prior risk-taking and its neural correlates on a subsequent decision. This study revealed several findings that were, at first glance, conflicting. However, put together, they suggest homeostatic activity of the IC (Figure 6). First, the researchers found increased dAIC activation during the feedback stage of risky prior experience trials, thus demonstrating that taking a risk elevated the dAIC activation. During the decision stage of probe trials they found slight deactivation in the dAIC following risk-taking in prior experience trials, and increased activation following non-risk prior decision trials, and that people took more risk following non-risk trials. Finally, increased activation of the bilateral vAIC during decision after non-risk led to increased risk-taking. Combining all these steps, we observe a seesaw-like fluctuation of the IC activity: it increases after risk-taking, moderates a bit during the following decision, which in turn leads to a safer choice, which would likely result in the urge to take risk, and thus increases again during yet another following decision that would likely result in risk taking.

We see evidence of the same dynamic in the Kuhnen and Knutson work that utilized an investment task in which participants had to choose between a bond and one of two stocks^11^. They found that stock selection (a risky choice) increased the likelihood of selecting a bond on a subsequent trial, which also correlated with increased activation in the AIC: “a 0.1% increase in anterior insula activation led to a 0.08% increase in the odds of choosing a bond” (Kuhnen and Knutson, 2005, p. 765). Interestingly, this dynamic is not limited to decisions immediately following risk-taking. Similar results were found in a gambling loss-chasing game (Campbell-Meiklejohn et al., 2008). In each trial, participants had a choice of accepting a loss or gambling for a chance of loss recovery that could also result in doubling that loss. They found that the decision to quit was mediated by activation in the dAIC, together with dACC and striatum. Although the authors did not examine neural correlates of initial loss, Kuhnen and Knutson (2005) did find elevated AIC activation for loss trials. Taken together, these findings suggest that increased activation in the AIC due to loss, when high enough, results in a decision to quit (Figure 5). An earlier study on risk-taking also found increased activation in the AIC during risk-taking decisions in comparison to safe choice, and the degree of AIC activation was related to the probability of selecting the safe choice after a risk trial resulting in loss (Paulus et al., 2003). Moreover, right AIC activation had strong positive correlations with harm avoidance and neuroticism. This last finding is particularly interesting, for it provides evidence of a conscious risk-avoidance tendency related to increase in insular activation due to protective and homeostatic function.

## A Model Perspective

The generalized perspective we used in presenting the insula’s role in decision-making can be applied to specific behavioral models. Two of the most fundamental and widely used models are Expected Utility Theory (EU; Neumann and Morgenstern, 1944) and Prospect Theory (PT; Kahneman and Tversky, 1979). Both postulate the existence of a function defined by the utility of possible outcomes and their probability; and that decision-making under uncertainty is focused on maximizing the EV of this function. The difference is that EU assumes a fully rational agent and thus estimates a rationally optimal decision. PT relates better to real-life choices by introducing a subjective component, a reference point, and by postulating that gains and losses have different curves. The reference point allows one to equate the utility of losses and gains, with smaller losses usually corresponding to larger gains due to loss aversion (Tversky and Kahneman, 1991). We propose that the IC plays a significant role in estimating the EV function. We have reviewed evidence showing that dAIC tracks the magnitude of rewards, both dAIC and vAIC are involved in evaluating variance of rewards, and the vAIC is sensitive to skewness of the rewards distribution, all important in evaluating possible outcomes. The AIC’s function in estimating risk prediction and risk prediction error is important in evaluating outcome probabilities. In addition, significant individual differences in anticipatory dAIC activation predictive of loss-aversion errors (Kuhnen and Knutson, 2005) can be viewed as indirect evidence of IC’s role in setting the reference point.

## Concluding Remarks

The current literature clearly identifies three sub-regions of the IC; this paper investigates the roles of the IC components in the decision making process. We build upon functional parcellation work that identifies the functional focus of the IC’s components.

**Figure 7:**
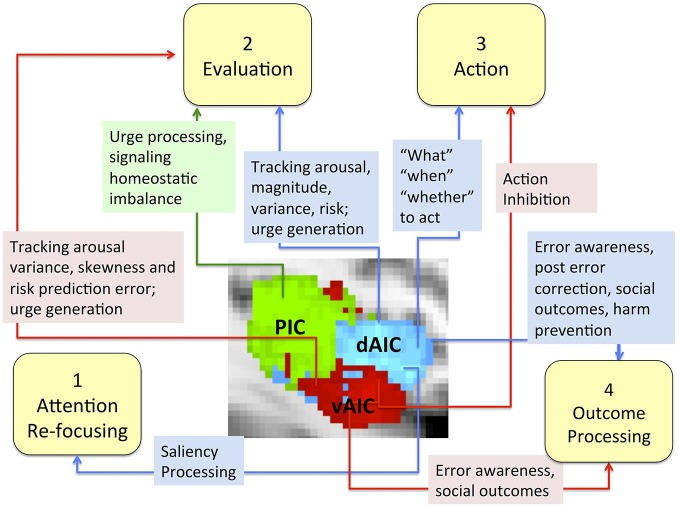
**The role of the IC and its sub-regions in each phase of decision-making.** Sub-regions of the Insula: posterior (green), dorsal anterior (blue) and ventral anterior (red).

More specifically, we conceptualized the decision making process as a sequence of four phases (focusing attention, evaluation, action and outcome processing) and discussed the role of the IC, particularly each of its components, in each phase (Figure 7). The evidence does not support any mapping of an IC subdivision onto a specific decision-making phase, but suggest the differences in the role each component plays. We discuss evidence that the dAIC, which seems to be functionally specialized for cognition and executive functioning, is critical in all four phases of the decision-making process. During the attention re-focusing phase, the dAIC and ACC form a SN responsible for identifying significant stimuli, recruiting processing resources such as the executive control network. During the evaluation phase, the dAIC is involved in tracking arousal, magnitude and risk, and it is also involved in urge generation. During the action selection phase, the dAIC is involved in choice and timing of actions, as well as in decisions as to whether to act or refrain from acting. During the outcome phase, the dAIC plays a role in processing errors and social outcomes, and it may be critical for harm prevention. The vAIC, on the other hand, is critical for emotional processing, chemo-sensation and autonomic function. It is also responsible for action inhibition during the action phase and error awareness and social outcome processing in the outcome phase. The vAIC also seems to be involved in a wide range of activities during the evaluation phase; specifically, urge generation and tracking arousal, variance skewness and risk prediction error. Finally, the evidence suggests that the PIC’s is involved only in the evaluation phase, where it is involved in urge processing and signaling homeostatic imbalance. This is consistent with its more general role in sensorimotor processing.

The model for dividing the neural correlates of decision-making into multiple phases has been highly influential in studying abnormal decision-making across many neuropsychiatric disorders (Ernst and Paulus, 2005). Our review suggests that although the IC subdivisions are closely connected and coordinated, each has a specific role in the decision-making process. The function of each of these sub-regions does not seem to be linked to one particular phase of the decision-making process, but rather may span all four phases as in the case of dAIC or be limited to one phase as per our current knowledge of PIC.

Finally, a review of the self-regulating and protective aspect of the IC functioning apparent in findings from the social and risky decision literature, suggested that actions like trust and risk-taking, which can be associated with potential danger, increase IC activation, which in turn attenuates use of such actions in future trials.

We also highlight the limitations in our understanding of the IC and it’s components’ role in decision-making that can now be overcome by current imaging methods and techniques. In many studies discussed in this review, we were unable to identify the different insula sub-regions involved in a particular function due to insufficient information. As the development of this research area on the insula and decision making progresses, researchers need to be more precise in identifying the links between a particular phase of the decision making process and the particular spatial sub-region of the insula when possible. This review highlights the increasing benefit of the ability to re-evaluate earlier findings in light of new knowledge and theories.

## Conflict of Interest Statement

The authors declare that the research was conducted in the absence of any commercial or financial relationships that could be construed as a potential conflict of interest.

## Footnotes

^1^NeuroSynth database contains thousands of automatically generated meta-analytical maps for each different term or topic. “Forward inference reflects the probability of observing activity in a region given knowledge of the psychological process” and reverse inference reflects “probability with which a given brain state implies a given mental state” (Chang et al., 2012; p. 741).

^2^Since only right insula was parcellated in this work, right insula mirror-image coordinates were used instead of the left insula coordinates for the remapping.

^3^Coordinates published in Talairax standard space in the original publications were first transformed to Montreal Neurological Institute (MNI) standard space with Signed Differential Mapping (SDM) coordinate converter utility (http://www.sdmproject.com/utilities/?show=Coordinates).

^4^Paulus and Frank (2006) identified dorsal anterior insula and Smith et al. (2009) found activation in ventral anterior insula.

^5^On the left side activation cluster also covers vAIC.

^6^Exception: Del Parigi et al. (2002) did not report the coordinates of increased activation.

^7^Electroencephalography.

^8^The paper identifies VLPFC as the area of interest, but the coordinates fall into dorsal anterior insula.

^9^The existence of such a network was previously suggested by Cosmides and Tooby (2000).

^10^No coordinates reported.

^11^Unfortunately this paper used an ROI defined based on activation in outcome stage so we can’t examine spatial differences in activation.
