# Supplementary material for: Roles of the Different Sub-Regions of the Insular Cortex in Various Phases of the Decision-Making Process
Source: Front Behav Neurosci. 2015 Nov 25;9:309. doi: 10.3389/fnbeh.2015.00309 (PMC4658437; doi:10.3389/fnbeh.2015.00309)
Supplement: Supplementary file 1 [file Table_1.pdf]

Table S1. MNI coordinates mapping in according with Chang et al. parcellations (2012). If original paper provided coordinates in Talairax space they are listed in the most right column.

Concept	Source	MNI Coordi-nates	Contrast	Insula Region	Talairax coordi-nates as reported
Self-initiated stopping	Brass & Haggard, 2007	-33,9,-12	stopping action vs. completing	vAI	-31,8,-6
Self-initiated stopping	Brass & Haggard, 2007	35,19,-17	stopping action vs. completing	vAI	32,17,-9
Urge to smoke PET & fMRI	Brody et al., 2002	38,23,-3	metabolic changes positively associated with urge to smoke in heavy smokers	dAI	34,20,4
Urge to smoke PET & fMRI	Brody et al., 2002	40,8,-2	metabolic changes positively associated with urge to smoke in heavy smokers	dAI	36,6,4
Urge to smoke PET & fMRI	Brody et al., 2002	-38,5,-7	metabolic changes positively associated with urge to smoke in heavy smokers	dAI	-36,4,-2
Urge to smoke PET & fMRI	Brody et al., 2002	-29,11,-12	metabolic changes positively associated with urge to smoke in heavy smokers	vAI	-28,10,-6
Urge to smoke	Brody et al., 2007	-49,17,1	positively correlated with craving	dAI	-46,14,6
Urge to smoke	Brody et al., 2007	-31,23,0	positively correlated with craving	dAI	-30,20,6
Urge to smoke	Brody et al., 2007	-40,13,2	positively correlated with craving	dAI	-38,10,6
Urge to smoke	Brody et al., 2007	-36,9,9	positively correlated with craving	dAI	-34,6,12
Skewness	Burke & Tobler, 2011	-33,14,-17	positive skewness vs. negative skewness	vAI	
Skewness	Burke & Tobler, 2011	-30,8,-17	positive skewness > 0 skewness > negative skewness	vAI	
Loss chasing game	Campbell-Meiklejohn et al., 2008	36,18,0	chasing losses vs. quit chasing losses	dAI	
Loss chasing game	Campbell-Meiklejohn et al., 2008	-32,20,2	chasing losses vs. quit chasing losses	dAI	
Connectivity	Cohen et al., 2005	-42,20,0	connectivity to ACC during high risk	dAI	
Connectivity	Cohen et al., 2005	35,25,-17	connectivity to ACC during low risk	vAI+[1]	
Cognitive Control Network	Cole & Schneider, 2007	38,22,5	target switching vs. non-switching	dAI	34,18,11
Cognitive Control Network	Cole & Schneider, 2007	-35,21,4	target switching vs. non-switching	dAI	-33,18,9
Task-set system	Dosenbach et al., 2006	-37,17,0	overlap of start-cue, sustained, and error-related activations	dAI	-35,14,5

[1] Plus sign indicates that the foci outside of the insula as defined by the masks but only 1 pixel off the area identified. Which means that the activation defined in the paper would include the identified area.

Concept	Source	MNI Coordinates	Contrast	Insula Region	Talairax coordinates as reported
Task-set system	Dosenbach et al., 2006	40,19,-3	overlap of start-cue, sustained, and error-related activations	dAI	36,16,4
Task-set system	Dosenbach et al., 2006	39,20,-2	start-cue processing attention re-focus	dAI	35,17,5
Task-set system	Dosenbach et al., 2006	-37,16,0	start-cue processing attention re-focus	dAI	-35,13,5
Neural correlates of consciousness of thirst	Egan et al., 2003	-39,-20,18	thirst vs. baseline 25min after saline injection	PI	-37,-22,18
Neural correlates of consciousness of thirst	Egan et al., 2003	43,19,6	maximum thirst increase	dAI	39,15,12
Neural correlates of consciousness of thirst	Egan et al., 2003	-41,27,-3	maximum thirst increase	dAI	-39,24,3
Neural correlates of consciousness of thirst	Egan et al., 2003	-41,15,8	maximum thirst increase	dAI	-39,12,12
Neural response to reward	Elliott et al., 2003	50,-11,-4	reward vs. no-reward	PI+	45,-12,0
Smoking cue reactivity	Engelman et al., 2012	-28,-26,14	ALE: smoking cue > neutral cue	vAI	-27,-27,14
Cocaine urges in users and controls	Garavan et al., 2000	45,-4,14	cocaine users exposed to cocaine film	PI	40,-7,17
Exposure to erotic stimuli	Gizewski et al., 2006	-44,-4,3	male-female in luteal phase	dAI	-42,-6,6
Exposure to erotic stimuli	Gizewski et al., 2006	50,-6,-6	male-female in luteal phase	PI+	45,-7,-1
Exposure to erotic stimuli	Gizewski et al., 2006	-44,-2,0	male-female menstrual phase	dAI	-42,-3,3
Exposure to erotic stimuli	Gizewski et al., 2006	48,-2,-2	male-female menstrual phase	ADI	43,-4,3
Exposure to erotic stimuli	Gizewski et al., 2006	-44,-2,-7	female in luteal vs. menstrual phase	PI+/dAI+	-42,-3,-3
Exposure to erotic stimuli	Gizewski et al., 2006	46,2,-7	female in luteal vs. menstrual phase	PI	42,0,-1
Error awareness	Harsay et al., 2012	34,18,-12	aware errors vs. unaware errors	vAI	
Error awareness	Harsay et al., 2012	50,8,-4	aware errors vs. unaware errors	dAI	
Anticipation of ambiguities uncertain gain	Huettel, 2006	41,19,9	decision phase, risk including ambiguity	dAI	
Internally triggered action when	Jenkins et al., 2000	-38,13,8	self initiated vs. externally triggered action	dAI	-36,10,12
Internally triggered action when	Jenkins et al., 2000	38,22,2	self initiated vs. externally triggered action	dAI	34,18,8
Drug related urges in men: cocaine	Kilts et al., 2001	-33,-2,15	cocaine imagery vs. neutral story	PI	-32,-5,17
Drug related urges in men: cocaine	Kilts et al., 2001	-41,21,0	cocaine imagery vs. neutral story	dAI	-39,18,5
Drug related urges in men: cocaine	Kilts et al., 2001	50,19,-7	cocaine imagery vs. anger story	dAI	45,16,0

Concept	Source	MNI Coordi-nates	Contrast	Insula Region	Talairax coordi-nates as reported
Drug related urges in men: cocaine	Kilts et al., 2001	-36,23,-6	cocaine imagery vs. anger story	dAI	-34,20,0
Drug related urges in women: cocaine	Kilts et al., 2004	-36,-1,6	cocaine images vs. neutral	PI	-34,-3,9
Error awareness	Klein et al., 2007	41,23,-14	aware errors vs. unaware errors	dAI+/ vAI +	37,21,-6
Error awareness	Klein et al., 2007	-43,14,-5	aware errors vs. unaware errors	dAI	-41,12,0
Anticipatory response to uncertain gain/loss	Knutson & Greer, 2008	33,23,-5	gain anticipation-loss anticipation	vAI	30,20,2
Anticipatory response to uncertain gain/loss	Knutson & Greer, 2008	27,23,-7	loss anticipation-gain anticipation	vAI	24,20,-8
Anticipatory response to uncertain gain/loss	Knutson & Greer, 2008	-38,19,-4	loss anticipation-gain anticipation	dAI	-36,16,2
Anticipatory response to uncertain gain/loss	Knutson & Greer, 2008	36,21,-7	gain anticipation -gain outcome	dAI	32,18,0
Anticipatory response to uncertain gain/loss	Knutson & Greer, 2008	51,14,-9	gain anticipation-gain outcome	dAI	46,12,-2
Monetary incentive delay	Knutson et al., 2000	37,19,0	reward trials vs. no incentive	dAI	33,16,6
Monetary incentive delay	Knutson et al., 2000	36,23,-3	punishment vs. no incentive	dAI	
Anticipatory response to predict purchase	Knutson et al., 2007	32,24,-9	viewing product, then price	vAI	29,21,-1
Anticipatory response to predict purchase	Knutson et al., 2007	-28,23,-10	viewing product, then price	vAI	-27, 21,-3
Risk vs. safe choice in investment paradigm	Kuhen & Knutson, 2005	-41,22,2	anticipatory	dAI	−39, 19, 7
Risk vs. safe choice in investment paradigm	Kuhen & Knutson, 2005	42,23,5	anticipatory	dAI	38, 19, 11
Self-initiated stopping	Kuhn & Brass, 2009	−39,21,−7	decide nogo vs. instructed stop	dAI	
Self-initiated stopping	Kuhn & Brass, 2009	42,25,-4	decide nogo vs. instructed stop	dAI	
SMH	Lawrence et al 2009	–36, 6, –6	deck selection in IGT: risky-safe	dAI	
SMH	Lawrence et al 2009	-30, 12,-12	deck selection in IGT: risky-safe	vAI	
SMH	Lawrence et al 2009	36,27,3	deck selection in IGT: risky-safe	dAI	
SMH	Lawrence et al 2009	-36, 15, -6	positive correlation with game score	dAI	
Post-error slowing	Li et al., 2008	36,20,8	post error trials slower RT vs. not no slower RT	dAI	

Concept	Source	MNI Coordi-nates	Contrast	Insula Region	Talairax coordi-nates as reported
Post-error slowing	Li et al., 2008	44,24,-4	post error trials slower RT vs. not slower RT	dAI	
Post-error slowing	Li et al., 2008	-32,20,12	post error trials slower RT vs. not slower RT	dAI	
Post-error slowing	Li et al., 2008	-44,16,-4	post error trials slower RT vs. not slower RT	dAI	
Urge to smoke	McBride et al., 2006	-36,12,2	smoking video vs. control video	dAI	
Risk components: value, perceived risk & risk attitude	Mohr et al., 2010	44,22,-2	making judgment of perceived risk	dAI	
Risk components: value, perceived risk & risk attitude	Mohr et al., 2010	32,12,-22	making judgment of perceived risk	vAI	
Internally triggered action what	Mueller et al.,2007	-41,7,2	which button to press: free choice vs. instruction	dAI	-39,5,6
Internally triggered action what	Mueller et al.,2007	43,10,-6	which button to press: free choice vs. instruction	vAI	39,8,0
Activation during alcohol cues in alcoholics	Myrick et al.,2004	41,-5,-14	alcohol vs. non alcoholic beverage	vAI	37,-5,-8
Activation during alcohol cues in alcoholics	Myrick et al.,2004	-48,15,-10	alcohol vs. non alcoholic beverage	dAI	-45,13,-4
Activation during alcohol cues in alcoholics	Myrick et al.,2004	47,22,-13	alcohol vs. control image	dAI+	43,20,-5
Activation during alcohol cues in alcoholics	Myrick et al., 2004	-39,3,-8	alcohol vs. control image	vAI	-37,2,-3
Task-level control	Nelson et al., 2010	-28,24,-18	Ventral ROI	vAI +	-27,22,-10
Task-level control	Nelson et al., 2010	-33,31,-8	Anterior ROI	vAI +	-31,28,-1
Task-level control	Nelson et al., 2010	-35,26,4	Dorsal ROI	vAI [2]	-33,22,9
Task-level control	Nelson et al., 2010	-34,17,9	Dorsal-posterior ROI	dAI[2]	-33,13,13
Task-level control	Nelson et al., 2010	41,12,-5	Posterior ROI	dAI	-37,10,1
Sure smaller win vs. chance of larger win	Paulus & Frank, 2006	-38,19,-8	anticipation: high reward vs. low reward	dAI	-36,17,-2
Diff expected reward and risk	Preuschoff et al., 2006	-34,-4,9	immediate expected reward value	PI	-33,-6,11
Diff. expected reward and risk	Preuschoff et al., 2006	-27,21,-5	immediate expected reward value	vAI	-26,18,1
Diff. expected reward and risk	Preuschoff et al., 2006	-34,20,-5	immediate reward variance	vAI	-32,17,1
Diff. expected reward and risk	Preuschoff et al., 2006	38,16,-6	immediate reward variance	dAI	34,13,1

[2] Since only the most anterior portion of insula was examined in this paper the ROI names do not match with sub-regions we are discussing

Concept	Source	MNI Coordi-nates	Contrast	Insula Region	Talairax coordi-nates as reported
Diff. expected reward and risk	Preuschoff et al., 2006	-31,25,4	delayed reward variance	vAI	-30,21,9
Risk prediction and risk prediction error	Preuschoff et al., 2008	36,17,-11	risk prediction error (1s epoch after 1st card)	vAI	32,15,-3.3
Risk prediction and risk prediction error	Preuschoff et al., 2008	-33,16,-8	risk prediction error (1s epoch after 1st card)	vAI	-31,14,-2.4
Risk prediction and risk prediction error	Preuschoff et al., 2008	37,25,1	risk prediction (between cards 1 & 2, 2d epoch)	dAI	33,21,8
Risk prediction and risk prediction error	Preuschoff et al., 2008	-32,26,2	risk prediction (between cards 1 & 2, 2d epoch)	vAI/dAI+	-31,22,7.7
Anticipation vs. consumption for monetary and social rewards	Rademacher et al., 2010	-48,11,-8	monetary reward anticipation vs.no reward	dAI	-45,9,-3
Anticipation vs consumption for $ and social rewards	Rademacher et al., 2010	34,-32,22	social reward anticipation vs. no reward	PI+	30,-34,21
Social decisions PD game	Rilling et al., 2008a	39,13,-4	unreciprocated vs. reciprocated cooperation	dAI	35,11,2
Social decisions PD game	Rilling et al., 2008a	-34,16,-12	unreciprocated vs. reciprocated cooperation	vAI	-32,14,-6
Social decisions PD game	Rilling et al., 2008a	41,25,-8	unreciprocated vs. reciprocated cooperation	dAI	37,22,0
Social decisions PD game	Rilling et al., 2008a	-34,19,-10	unreciprocated vs. reciprocated cooperation	vAI	-32,17,-4
Neural correlates of sexual arousal	Safron et al., 2007	-34,5,16	erotic preferred gender vs. non-preferred	PI	-33,2,18
Neural correlates of sexual arousal	Safron et al., 2007	-39,-1,1	erotic preferred gender vs. sports	PI	-37,-3,4
Cue reactivity in heroin addicts	Sell et al., 1999	-40,11,-14	after heroin injection while watching heroin salient video	vAI	-38,10,-8
Reward magnitude	Smith et al., 2009	42,-6,-10	Large - small reward	vAI	38,-7,-5
Salience Network	Sridharan et al., 2008	34,26,-6	Insula ROI	dAI	
Neural coding of risk dimensions: skewness and variance	Symmonds et al., 2011	30,16,-14	positive correlation with increase in positive skewness	vAI	
Cue reactivity in alcohol dependent vs. controls	Tapert et al., 2004	-47,-11,15	alcohol cues, alcohol dependent vs. control participants	PI	-45,-13,16
Neural correlates of hunger and satiation	Tataranni et al., 1999	-44,18,-6	hunger after 36hr fast vs. satiation (liquid meal)	dAI	-42,16,0
Neural correlates of hunger and satiation	Tataranni et al., 1999	38,-7,-5	hunger after 36hr fast vs. satiation (liquid meal)	PI	34,-8,0
Itching and scratching	Vierow et al., 2009	-37,16,2	scratching during itch	dAI	-35,13,7
Itching and scratching	Vierow et al., 2009	45,16,-4	scratching during itch	dAI	41,13,3

Concept	Source	MNI Coordi-nates	Contrast	Insula Region	Talairax coordi-nates as reported
Itching and scratching	Vierow et al., 2009	49,-27,21	scratching during itch	in paper PI[3]	44,-29,21
Itching and scratching	Vierow et al., 2009	-48,-32,23	scratching during itch	in paper PI[3]	-46,-33,21
Uncertainty	Volz, 2004	-27,28,0	Anticipation of uncertainty vs. certainty	vAI	-26,24,6
Uncertainty	Volz, 2004	31,26,2	Anticipation of uncertainty vs. certainty	vAI	28,22,9
Skewness	Wu et al., 2011	33,27,-7	High vs low variance	vAI	30, 24, 1
Skewness	Wu et al., 2011	--32,26,-11	High vs low variance	vAI +	-30, 24, -4
Skewness	Wu et al., 2011	33,26,-7	Skew vs. non skew	vAI	30, 23, 1
Skewness	Wu et al., 2011	-35,26,-5	Skew vs. non skew	vAI	-33, 23, 1
ALE meta on mean, variance and skewness	Wu et al., 2012	-34,18,-6	High vs. low variance	vAI	-32,16,0
ALE meta on mean, variance and skewness	Wu et al., 2012	36,16,-9	High vs. low variance	vAI	-32,14,-2
ALE meta on mean, variance and skewness	Wu et al., 2012	36,19,-5	High vs. low mean	dAI	34,16,2
ALE meta on mean, variance and skewness	Wu et al., 2012	-31,20,-6	High vs. low mean	vAI	-30,18,0
Effect of prior risk	Xue et al., 2010	-36,14,-2	outcome phase, risk vs. no-risk	dAI	
Effect of prior risk	Xue et al., 2010	-38,14,-2	outcome phase, risk vs. no-risk	dAI	
Effect of prior risk	Xue et al., 2010	40,18,-10	outcome phase, risk-win vs. no-risk	dAI	
Effect of prior risk	Xue et al., 2010	-34,10,8	decision after non-risk vs. risk win	dAI	
Effect of prior risk	Xue et al., 2010	48,4,2	decision after non-risk vs. risk win	dAI	
Effect of prior risk	Xue et al., 2010	48,2,0	risk vs. non-risk decisions	dAI	
Effect of prior risk	Xue et al., 2010	34,22,-14	risk-seeking after non-risk vs. after risk-win	vAI	
Effect of prior risk	Xue et al., 2010	-30,24,2	risk-seeking after non-risk vs. after risk-win	vAI	

[3] The coordinates fell outside of the mask but the area identified in paper as stated (PI)
