# Supplementary material for: Roles of the Different Sub-Regions of the Insular Cortex in Various Phases of the Decision-Making Process
Source: Front Behav Neurosci. 2015 Nov 25;9:309. doi: 10.3389/fnbeh.2015.00309 (PMC4658437; doi:10.3389/fnbeh.2015.00309)
Supplement: Supplementary file 2 [file Table_2.pdf]

Table S2. Glossary

Abbreviation	Term
ACC	Anterior Cingulate Cortex
AIC	Anterior Insular Cortex
CCN	Cognitive Control Network
CEN	Central Executive Network
dACC	dorsal Anterior Cingulate Cortex
dAI	dorsal Anterior Insula
dAIC	dorsal Anterior Insular Cortex
DLPFC	Dorsolateral Prefrontal Cortex
DMN	Default Mode Network
dPMC	dorsal Pre-Motor Cortex
EEG	Electroencephalography
IAPS	Affective Picture System
IC	Insular cortex
IFJ	Inferior Frontal Junction
IGT	Iowa Gambling Task
LOFC	Lateral Orbitofrontal Cortex
MPFC	Medial Prefrontal Cortex
Nacc	Nucleus Accumbens
OFC	Orbitofrontal Cortex
PFC	Prefrontal Cortex
PI	Posterior Insula
PIC	Posterior Insular Cortex
pMFC	Posterior Medial Frontal cortex
PPC	Posterior Parietal Cortex
pSMA	pre-Supplementary Motor Area
SMA	Supplementary Motor Area
SMH	Somatic Marker Hypothesis
TPJ	Temporo-Parietal Junction
vAI	Ventral Anterior Insula
vAIC	Ventral Anterior Insular Cortex
VTA	Ventral-Tegmental Area
